# Understanding the Metabolic and Endocrine Effects of Macronutrient Boluses in the Context of a Low-Carbohydrate Diet: A Randomized Crossover Study

**DOI:** 10.3390/nu18162669

**Published:** 2026-08-15

**Authors:** Landon S. Deru, Bruce W. Bailey, Katelynn E. Hales, Elizabeth Z. Gipson, Ray M. Merrill, Benjamin T. Bikman

**Affiliations:** 1 Department of Internal Medicine, The University of Kansas Medical Center, Kansas City, KS 66160, USA; 2 Department of Exercise Sciences, Brigham Young University, Provo, UT 84602, USA; 3 Department of Public Health, Brigham Young University, Provo, UT 84602, USA; 4 Department of Cell Biology and Physiology, Brigham Young University, Provo, UT 84602, USA

**Keywords:** low-carbohydrate diet, blood glucose, metabolic health, glycemic control

## Abstract

**Background/Objectives**: Adherence to a low-carbohydrate diet induces distinct metabolic adaptations, yet the acute endocrine responses to individual macronutrients within this adapted state remain incompletely characterized. This study aimed to determine the acute, postprandial endocrine and glycemic kinetics following isolated isocaloric carbohydrate, protein, and fat boluses in healthy adults adapted to a low-carbohydrate diet. **Methods**: Participants (13 female, 11 male) completed three interventions which consisted of consuming an isovolumetric 300 kcal drink of either whey protein (PRO), dextrose (CHO), or olive oil (FAT) mixed with water. Blood was taken immediately prior to drink consumption and then every 30 min afterwards for 2 h in each condition. Continuous glucose monitoring occurred during each intervention. **Results**: Postprandial glucose remained stable following the FAT and PRO conditions, whereas the CHO condition significantly increased glucose concentrations over time (*p* < 0.01). Glucagon did not differ between FAT and CHO (*p* = 0.28) but increased following PRO compared to both FAT and CHO (*p* < 0.01). Insulin, C-peptide and amylin rose in the CHO condition, with smaller increases in PRO and minimal changes in FAT (*p* < 0.01). GLP-1 was elevated in the PRO condition compared to both FAT and CHO (*p* < 0.01), while GIP increased most in the CHO condition and differed across all treatments (*p* < 0.01). Ghrelin decreased after CHO and PRO, leptin did not significantly change across conditions, and both PP and PYY were lower in the CHO condition at later time points relative to FAT and PRO. Inflammatory markers showed minimal acute changes across conditions, although MCP-1 increased over time following PRO. **Conclusions**: In low-carbohydrate-adapted individuals, isolated macronutrient boluses elicit highly distinct, divergent endocrine signatures. The robust, acute glucagon response to pure protein does not cause short-term glycemic excursions, indicating that counter-regulatory incretin and insulinotropic pathways remain highly capable of preserving immediate glucose homeostasis.

## 1. Introduction

Diabetes remains a major and growing global health burden. Recent pooled analyses from the NCD Risk Factor Collaboration estimated that the number of adults living with diabetes increased substantially from 1990 to 2022, reaching approximately 828 million adults worldwide by 2022 [1]. In the United States, type 2 diabetes (T2D) and its precursors, including prediabetes and insulin resistance, remain among the most common metabolic disorders [2]. The primary measure of both diagnosis and therapeutic treatment for these disorders is blood glucose concentrations [3]. Unfortunately, this fixation on glucose alone has resulted in a collective oversight of the hormones that regulate it, namely insulin, glucagon and incretin hormones.

In healthy individuals, insulin performs many roles, including the uptake of glucose into skeletal muscle and the inhibition of hepatic gluconeogenesis [4]. Individuals who become insulin resistant often experience blood lipid dysregulation, an increase in inflammatory cytokines, and mitochondrial dysfunction [5]. In this state, the body also handles an overabundance of glucagon, which, via hepatic gluconeogenesis and glycogenolysis, antagonizes insulin’s efforts and applies an upward pressure on rising blood glucose. Thus, in T2D and IR, both insulin and glucagon are elevated [6].

Beyond its relevance in chronic insulin resistance, characterizing the acute response to individual macronutrients provides critical insight into the metabolic plasticity of individuals adapted to carbohydrate restriction. While a low-carbohydrate diet shifts the primary substrate utilization toward lipid oxidation, the acute regulatory response to individual metabolic challenges—such as pure glucose, amino acids, or fatty acids—remains poorly understood in this physiological state. For instance, dietary protein is a potent stimulus of glucagon secretion, and it has been hypothesized that a high habitual protein intake could alter basal or postprandial glucagon kinetics, potentially affecting hepatic glucose output. However, the precise coordination of pancreatic, incretin, and appetite-regulating hormones in response to isolated macronutrient boluses has not been comprehensively mapped within a low-carbohydrate-adapted cohort.

The purpose of this study was to characterize the acute, multi-system endocrine and glycemic profiles following the ingestion of isolated, isocaloric boluses of carbohydrate, protein, and fat in adults maintaining a low-carbohydrate diet. Specifically, we sought to determine how these isolated nutritional challenges modulate the immediate network of glycemic control, appetite, and inflammatory signaling molecules within a 120 min postprandial window.

## 2. Materials and Methods

Using a randomized crossover design with counterbalanced treatment conditions, participants were enrolled in the study for a duration of 4 weeks. Baseline testing took place during the first week and the three intervention conditions took place from weeks 2 to 4. Participants completed all three intervention arms with a 6- to 10-day inter-session recovery and clearance period between each intervention session to ensure that the acute, short-term metabolic and physiological effects of the specific macronutrient bolus administered during each testing session had completely dissipated before the second session. The order of conditions was randomly determined (randomizer.org) and assigned to participant numbers prior to the start of the study. Participant numbers were given sequentially based on the order in which they signed the consent form and officially enrolled in the study. Before each intervention session, participants were screened for contraindications to ensure continued eligibility. Approval from the university’s Institutional Review Board was obtained prior to initiating any aspect of this study. This study was retrospectively registered at ClinicalTrials.gov following study completion because this study was not a general clinical medical study; the local authorities did not require prospective trial registration before the trial began. The study protocol, eligibility criteria, intervention, and planned outcomes were established before participant enrollment and were not modified at trial registration. This study was registered at https://clinicaltrials.gov/study/NCT07713316. (Registration code: NCT07713316). Registration date: 20 July 2026.

### 2.1. Participants

Participants were between 18 and 65 years of age and were weight stable (±3% body weight) for the past 3 months. Additionally, participants must have been adhering to a low-carbohydrate (50 g carbohydrates or less per day) diet at least 2 weeks prior to and during the duration of the one-month study. The requirements for inclusion were vetted through the initial qualification screening and further confirmed verbally at the initial screening appointment. Individuals interested in the study were sent a qualification survey (Qualtrics.com) before enrollment to determine participation eligibility. Potential participants were excluded if they did not provide written consent for participation or if they met any of the following criteria:

1. Diagnosed with chronic disease(s) including cardiac disease, cancer, or liver disease;
2. Pregnant or lactating;
3. Taking medications including anticoagulants and those that may alter metabolism (i.e., insulin, metformin, and amphetamine-based ADHD medications);
4. Had allergies to latex, cow’s milk/milk products, olives, chlorhexidine, or dextrose;
5. Had more than 5 X-rays within the past year.

### 2.2. Measurements

Blood pressure and heart rate were taken using an automated sphygmomanometer (Omron Inc., Kyoto, Japan, Model HEM-907XL) at each visit prior to phlebotomy to ensure safety. If the systolic pressure was less than 100 mmHg or if the diastolic pressure was below 60 mmHg, phlebotomy was not performed. No participants were excluded or rescheduled based on these blood pressure criteria.

#### 2.2.1. Anthropometric and Body Composition Measures

Body weight and height were measured for all participants at the beginning of each session. Weight was measured using a digital scale (Seca, Hamburg, Germany) accurate to ±0.1 kg with participants dressed in athletic shorts and a t-shirt and with shoes removed. Height was measured with shoes removed by a stadiometer accurate to ±0.1 cm (Seca, Hamburg, Germany). Body mass index (BMI) was calculated as weight (in kilograms) divided by the square of height (in meters). A GE iDXA (GE, Fairfield, CT, USA) was used to assess fat-free mass, fat mass, lean mass, percent body fat and visceral adipose tissue [7]. Calibration of the dual-energy X-ray absorptiometer (DXA) scanner took place at the beginning of each testing day using a manufacturer-provided calibration block. Scans were analyzed using Encore software version 17.

#### 2.2.2. Plasma Hormone Concentrations

The Human Metabolic Hormone Magnetic Bead Panel multiplex kit (Sigma-Aldrich, Inc., St. Louis, MO, USA; Catalog # HMHEMAG-34K) was utilized to quantify the plasma concentrations of 13 metabolic hormones, including amylin, ghrelin, glucagon, glucagon-like peptide 1 (GLP-1), glucose-dependent insulinotropic polypeptide (GIP), interleukin 6 (IL-6), insulin, c-peptide, leptin, monocyte chemoattractant protein-1 (MCP-1), pancreatic polypeptide (PP), peptide YY (PYY), and tumor necrosis factor alpha (TNF-α). Venipuncture took place in the human performance laboratory by a trained phlebotomist using standard phlebotomy procedures, including sterile technique, to ensure that risks to the participants were minimized. A single 4 mL vacuum-sealed tube prepared with Ethylenediaminetetraacetic acid (EDTA) was taken from each participant at or near the median cubital vein. Each 4 mL tube was inverted to allow for mixture with the EDTA and then samples were centrifuged for 15 min at 1500 rotations per minute within 10 min of collection, after which the plasma was aliquoted and placed into cryovials. Plasma samples were then stored at −80 °C for later analysis.

#### 2.2.3. Continuous Glucose Monitoring

Interstitial subcutaneous glucose concentrations were measured using the Freestyle Libre 2 continuous glucose monitor (CGM) (Abbott Laboratories, Abbott Park, IL, USA). The interstitial glucose concentration closely reflects the intravascular glucose concentration, making this a useful tool to measure the glycemic response to various stimuli [8]. Each CGM was inserted on the back of the non-dominant upper arm and given at least 60 min to calibrate, per manufacturer specifications, to ensure sensor accuracy [9]. This sensor recorded interstitial glucose concentrations every 15 min and these data were downloaded to the participants’ personal smartphones using the Freestyle Libre mobile application. These data were then uploaded to online cloud storage for later analysis.

### 2.3. Procedures

Once eligibility was determined and informed consent was provided, participants were sent a link provided by Levels Heath, Inc., to order a one-month supply (two units) of continuous glucose monitors (CGMs). Once the CGM units arrived in the mail, subjects scheduled their baseline assessment visit. For this visit and all intervention visits, subjects arrived at the human performance laboratory having fasted 8–12 h, maintained normal sleep patterns, and refrained from participating in strenuous exercise for the past 24 h. Adherence to the pre-test-day protocols was gauged at the beginning of each session. If the pre-test protocols had not been followed, the participant was rescheduled. DXA scan was completed to measure the body composition parameters of interest. Participants were scanned wearing loose clothing with no jewelry or items in their pockets and their shoes off. Following the DXA scan, subjects underwent a single blood draw (4 mL) and a continuous glucose monitor was placed and activated according to manufacturer recommendations.

#### Intervention Sessions

Following the baseline assessment, study subjects were scheduled for three intervention visits, spaced 6–10 days apart. At each intervention visit, subjects were screened for protocol adherence and one of three dietary conditions was initiated. The dietary conditions included a carbohydrate beverage, a protein beverage, and a fat beverage. Condition order was assigned randomly prior to the intervention visits for each participant. The carbohydrate beverage consisted of 300 kcal (88 g) of dextrose powder dissolved in 12 oz of water. The protein beverage consisted of 300 kcal (45 g) of unflavored whey powder dissolved in 12 oz in water. The fat beverage consisted of 300 kcal (1.25 oz) of olive oil suspended in 12 oz of water. While blinding the participants to the beverage type was attempted, the impact was likely minimal due to the palatability and taste of each drink which immediately informed them of the drink type.

At each session, venous blood samples were taken at 0 min, 30 min, 60 min, 90 min, and 120 min. Immediately following the first blood draw (0 min), the participant consumed the entire assigned beverage within 5 min. Between draws, participants were allowed to rest, either sitting or slowly moving about the room, but were asked to refrain from eating or excessive movement. At the conclusion of each visit, the participant was excused from the laboratory and returned on their assigned days to complete the remaining conditions of the study.

### 2.4. Statistical Techniques

An a priori sample size was estimated based on a β-1 of 0.8, an alpha of 0.05, and a moderate effect size (d = 0.66) to detect a 30% difference in glucose area under the curve between conditions. From these calculations, it was determined that a total of 22 participants were needed for adequate statistical power. Twenty-four participants were ultimately recruited anticipating that some individuals would be lost to attrition.

The data were described using frequencies, percentages, and standard deviations. Prior to analysis, each variable was assessed for normality. Mixed-effects models were used to evaluate postprandial hormonal responses using the PROC MIXED procedure. Each hormone (glucagon, insulin, C-peptide, amylin, ghrelin, GIP, GLP-1, pancreatic polypeptide, PYY, TNF-α, IL-6, MCP-1, and leptin) was analyzed separately with condition (carbohydrate, fat, protein), time, and their interaction as fixed effects and participant as a random effect. Body mass index (BMI) was retained as a continuous fixed covariate in all final mixed-effects models to control for the potential confounding effects of baseline adiposity on endocrine and metabolic kinetics. Biological sex was initially included but removed from final models as it exerted no significant main or interactive effects on any outcome variable (*p* > 0.25). Post hoc multiple pairwise comparisons for significant main or interactive effects were performed using the LSMEANS statement. Repeated measures across time were modeled using several covariance structures, including compound symmetry (CS), unstructured, and first-order autoregressive structures. Model fit indices (AIC, AICC, and BIC) were compared across structures, and CS provided the best overall fit; therefore, CS was used for all reported analyses. Degrees of freedom were estimated using the Kenward–Roger method. Significant condition × time interactions were further examined using prespecified pairwise contrasts evaluating (1) changes from baseline within each condition and (2) differences between conditions at each postprandial time point. Effect sizes for omnibus mixed-model effects were reported as partial eta-squared (η^2^p). Effect sizes for pairwise comparisons were calculated as Cohen’s dz for repeated measures using the mean and standard deviation of the paired difference scores. Statistical significance was set at *p* < 0.05. Data were evaluated using the statistical software package (Base SAS 9.4; SAS Institute, Inc., Cary, NC, USA, 2016).

## 3. Results

**Figure 1:**
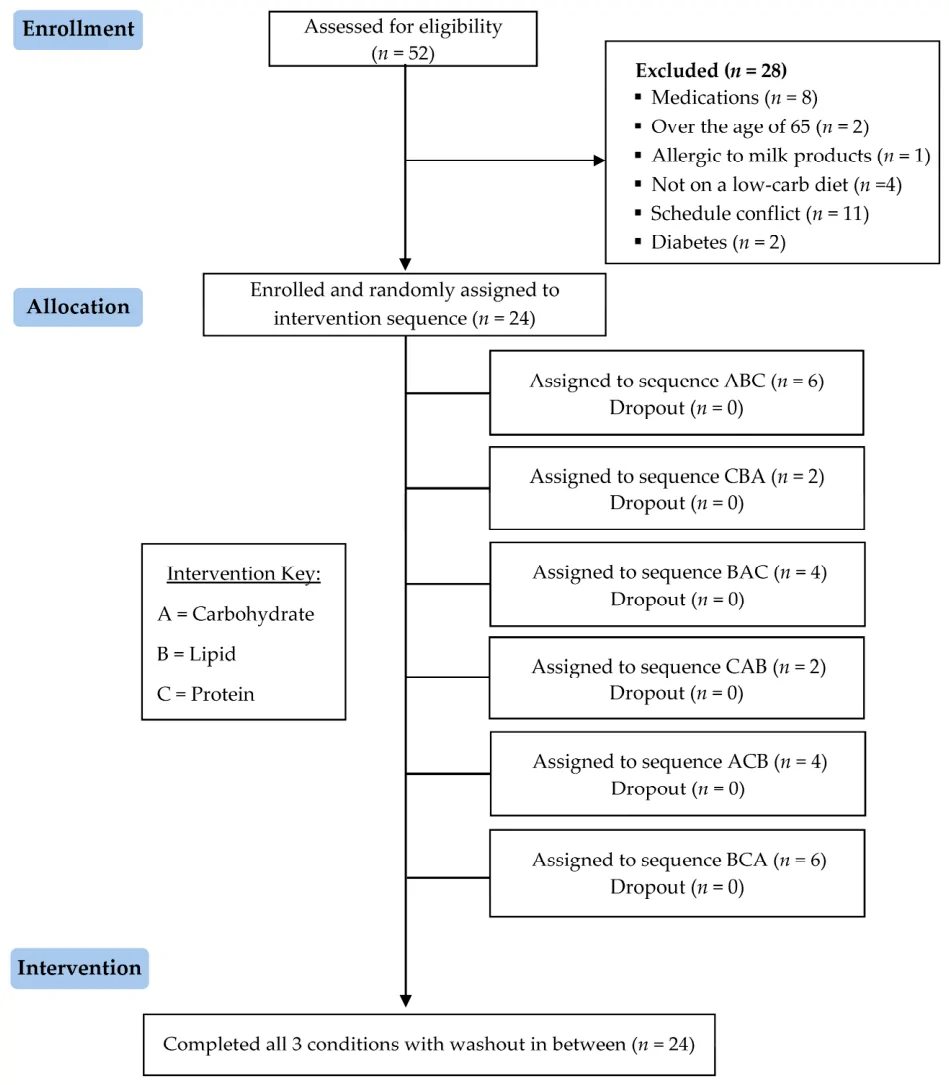
Participant flow diagram.

Table 1 presents the demographic characteristics of the sample for this study. Of the 52 individuals who completed the qualification survey, 24 (14 females) qualified, enrolled, and consented to participate in the study (Figure 1). Of the 24 who completed the study, 16 (8 females) had viable data from continuous glucose monitors. There was not a significant difference in the distribution of gender with the larger sample (chi-square *p* = 0.5212). Gender was applied in the model, but it did not have a significant effect on any of the outcome variables and was removed. The ages of the participants ranged from 22 to 59 with no significant difference in distribution (*p* = 0.8003). The body mass index (BMI) of the participants ranged from 21.4 to 44.6 with no significant difference in distribution (*p* = 0.4341).

1. Continuous Glucose Monitoring Outcomes

Of the 24 participants who completed the study, 16 had viable data from the continuous glucose monitors. The remaining 8 participants experienced complete or partial data loss due to unexpected sensor technical failures (*n* = 1), premature sensor dislodgement (*n* = 3), or mobile application synchronization errors (*n* = 4). Independent-samples *t*-tests and chi-square analyses revealed that the subgroup with non-viable CGM data did not differ significantly from the viable subgroup in age (*p* = 0.68), BMI (*p* = 0.54), sex distribution (*p* = 0.72), or baseline plasma hormone concentrations (all *p* > 0.05). Interstitial glucose monitoring spanned from 60 min prior to ingestion (−60 min) to 240 min post-ingestion (+240 min) to fully capture the preprandial steady-state and late-phase glycemic recovery under free-living conditions surrounding the acute 120 min laboratory blood collection window.

**Figure 2:**
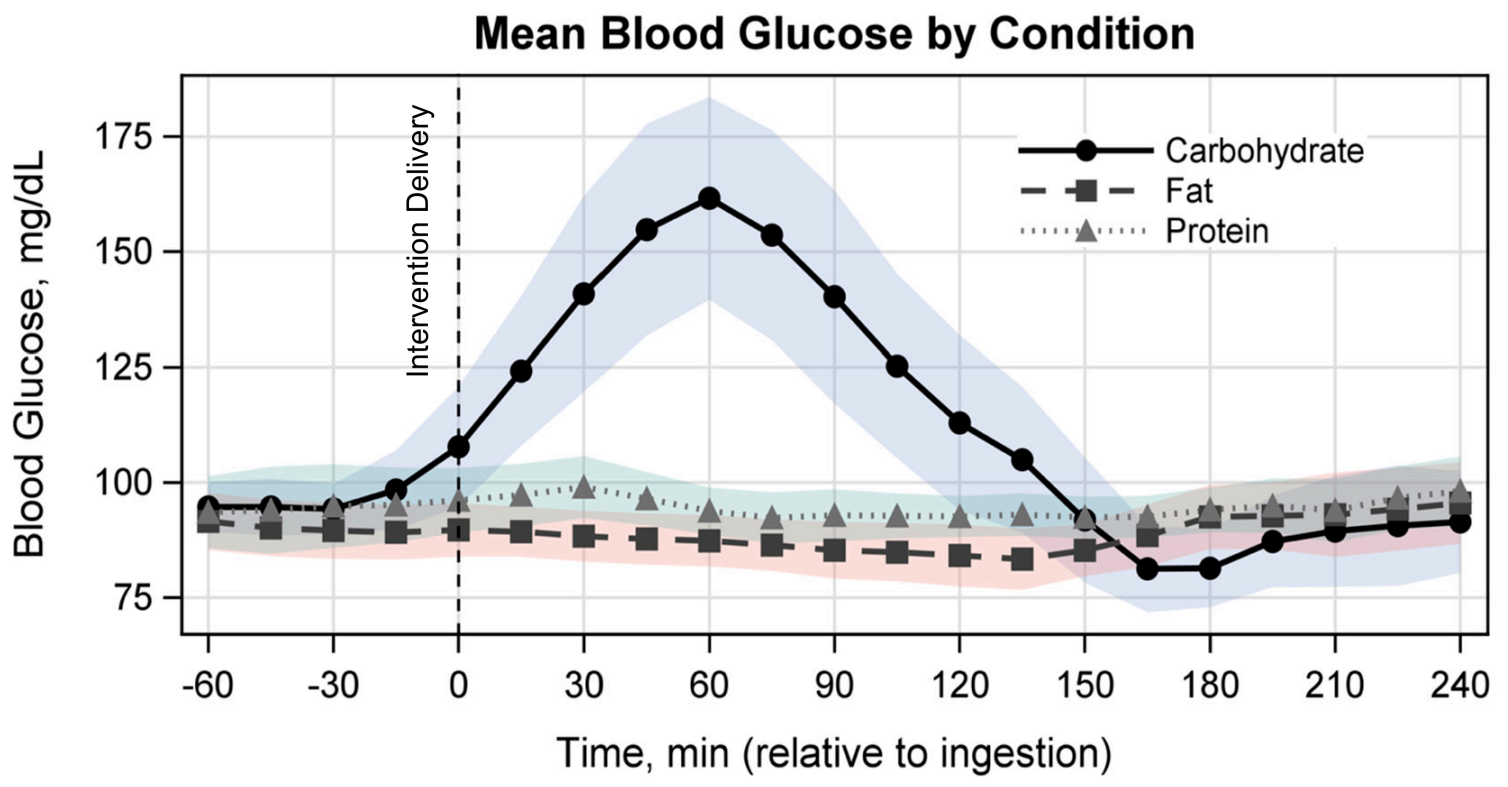
Concentrations of interstitial glucose (from continuous glucose monitor) over time. Shaded band: 95% CI.

Postprandial glucose responses differed markedly by macronutrient (Figure 2) with a significant time effect across all three conditions (F = 7.60, *p* < 0.0001). Carbohydrate ingestion produced a rapid rise in glucose that peaked at 60 min before returning to baseline by 150 min. In contrast, glucose concentrations remained stable following both fat and protein ingestion and were not different to each other (95% CI = −5.31–16.52 *p* = 0.4367).

2. Plasma Hormone Outcomes

At baseline (Time 0), no significant differences were detected among the CHO, FAT, and PRO conditions for any of the 13 assessed hormones (all *p* > 0.50). The condition × time interaction was subsequently assessed for all hormones. Significant condition × time interactions were observed for insulin (*F*_8,268_ = 7.56, *ηp*^2^ = 0.18, *p* < 0.0001), glucagon (*F*_8,268_ = 18.71, *ηp*^2^ = 0.36, *p* < 0.0001), amylin (*F*_8,268_ = 18.85, *ηp*^2^ = 0.36, *p* < 0.0001), C-peptide (*F*_8,268_ = 15.51, *ηp*^2^ = 0.32, *p* < 0.0001), GIP (*F*_8,268_ = 8.46, *ηp*^2^ = 0.20, *p* < 0.0001), GLP-1 (*F*_8,268_ = 17.38, *ηp*^2^ = 0.34, *p* < 0.0001), ghrelin (*F*_8,268_ = 3.91, *ηp*^2^ = 0.11, *p* = 0.0002), PYY (*F*_8,268_ = 2.65, *ηp*^2^ = 0.07, *p* = 0.0082), and MCP-1 (*F*_8,269_ = 2.33, *ηp*^2^ = 0.07, *p* = 0.0194), indicating that meal-induced hormonal responses differed by condition. No significant condition × time interaction was detected for PP, leptin, TNF-α, or IL-6 (Table 2).

### 3.1. Glycemic Control Hormone Outcomes

**Figure 3:**
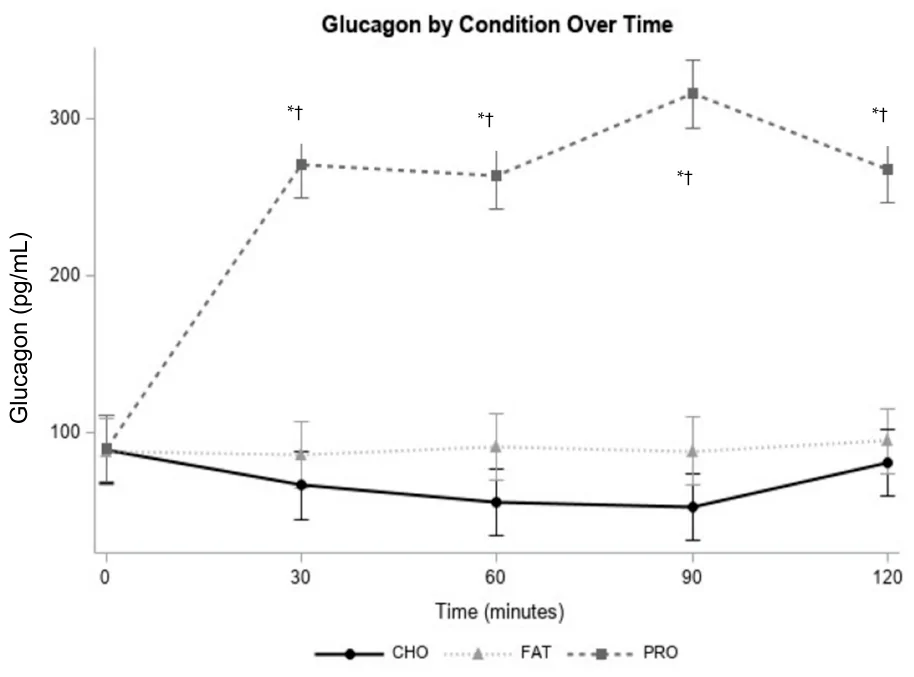
Concentrations of glucagon over time. * CHO significantly different than PRO (*p* < 0.05). † PRO significantly different than FAT (*p* < 0.05). Mean ± Standard Error.

Postprandial glucagon concentrations (Figure 3) did not differ significantly from baseline during the carbohydrate or fat conditions (*p*’s > 0.05). In contrast, glucagon increased rapidly following protein ingestion, rising from baseline over the initial 30 min (*t*_266_ = 10.16, *p* < 0.0001, *d*z = 1.22) and peaking at 90 min (*t*_266_ = 12.52, *p* < 0.0001, *d*z = 1.45). Concentrations declined by 120 min (*t*_266_ = 10.05, *p* < 0.0001, *d*z = 1.42) but remained elevated compared with the CHO (*d*z = 1.18) and FAT (*d*z = 1.30).

**Figure 4:**
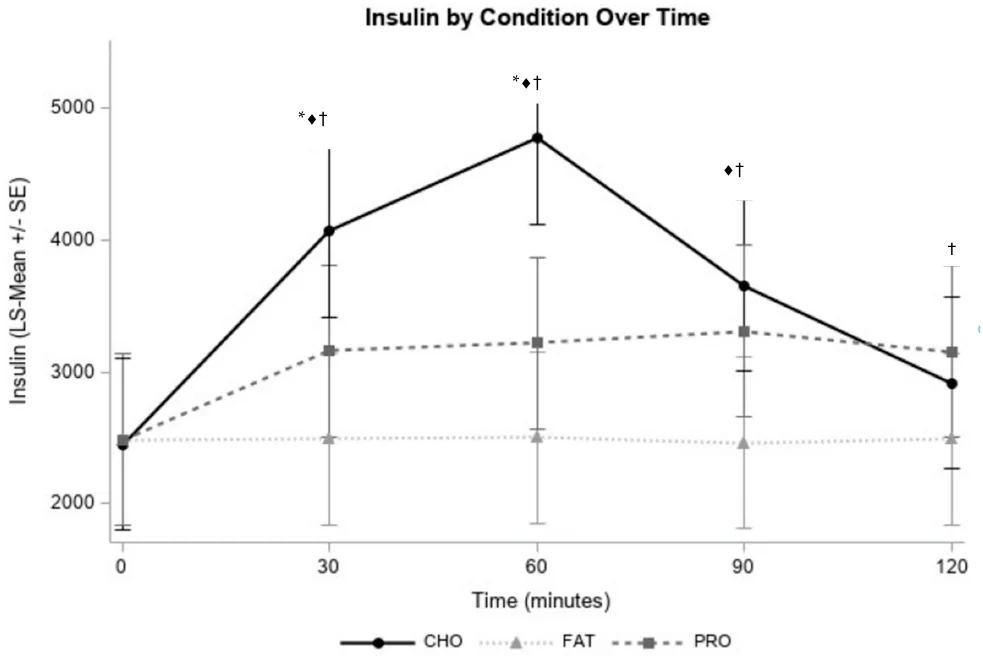
Concentrations of insulin over time. * CHO significantly different than PRO (*p* < 0.05). ♦ CHO significantly different than FAT (*p* < 0.05). † PRO significantly different than FAT (*p* < 0.05). Mean ± Standard Error.

For insulin (Figure 4), the CHO response was significantly higher than both FAT and PRO at 30 and 60 min. Compared with FAT, the magnitude of the difference was moderate at 30 min (*d*z = 0.66) and large at 60 min (*d*z = 1.25). Compared with PRO, the difference was small-to-moderate at 30 min (*d*z = 0.33) and moderate-to-large at 60 min (*d*z = 0.86) (all *p* ≤ 0.0031). By 120 min, insulin concentrations following CHO returned toward baseline and were no longer different from FAT (*t*_333_ = 1.59, *p* = 0.1138). In contrast, the PRO meal induced a sustained rise in insulin from 30 min onward and remained elevated relative to FAT at 120 min (*d*z = 1.16).

**Figure 5:**
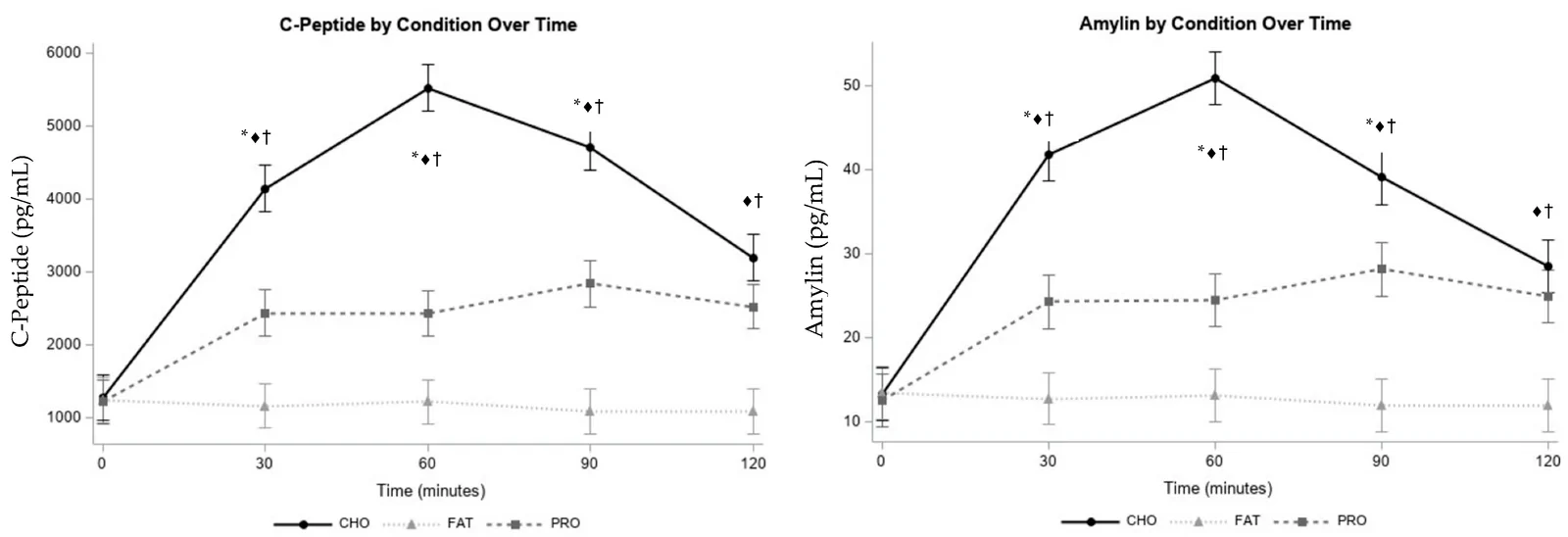
Concentrations of C-peptide and amylin over time. * CHO significantly different than PRO (*p* < 0.05). ♦ CHO significantly different than FAT (*p* < 0.05). † PRO significantly different than FAT (*p* < 0.05). Mean ± Standard Error.

A similar pattern was observed for C-peptide and amylin (Figure 5). Both hormones increased substantially following CHO and PRO ingestion and remained elevated relative to FAT throughout the postprandial period. The CHO response peaked at 60 min for both C-peptide (*d*z = 1.72) and amylin (*d*z = 1.83), whereas the PRO response peaked at 90 min for C-peptide (*d*z = 1.80) and amylin (*d*z = 1.66). At 120 min, concentrations remained higher in both CHO and PRO than FAT for C-peptide (*d*z = 1.32 and 1.56, respectively) and amylin (*d*z = 1.34 and 1.39, respectively). The CHO response exceeded the PRO response during the first 90 min, after which the two conditions converged.

**Figure 6:**
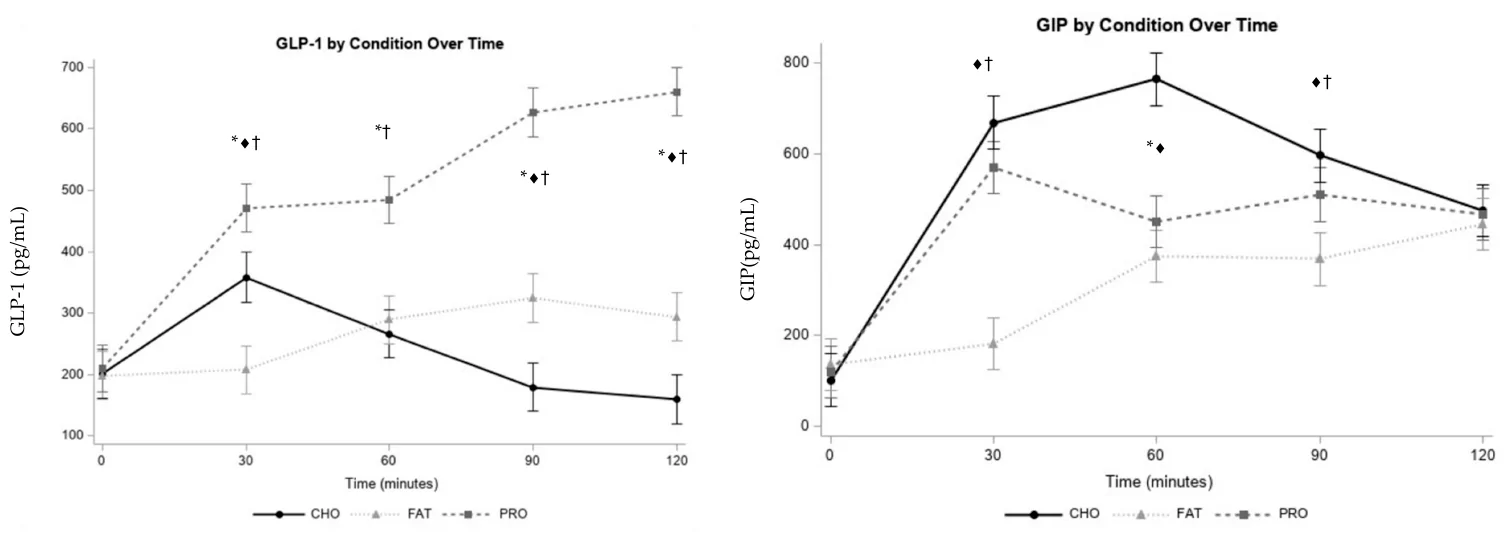
Concentrations of GLP-1 and GIP over time. * CHO significantly different than PRO (*p* < 0.05). ♦ CHO significantly different than FAT (*p* < 0.05). † PRO significantly different than FAT (*p* < 0.05). Mean ± Standard Error.

The postprandial profiles for the incretin hormones GIP and GLP-1 diverged significantly (Figure 6). For GIP, the CHO and PRO meals elicited a rapid, comparable rise after 30 min (*t*_179_ = 1.32, *p* = 0.1901, *d*z = 0.24). The CHO response then significantly exceeded that of PRO at 60 min (*t*_175_ = 4.26, *p* < 0.0001, *d*z = 0.91). The FAT meal showed a delayed GIP rise that became apparent at 60 min, although it was not statistically different from PRO at that time (*t*_175_ = 1.06, *p* = 0.2928, *d*z = 0.20). At 90 min, CHO concentrations remained significantly higher than those of FAT (*t*_179_ = 3.08, *p* = 0.0024, *d*z = 0.47). By 120 min, no significant pairwise differences remained among the conditions (all *p* > 0.9295).

In contrast, GLP-1 exhibited the largest overall response following the PRO meal, with concentrations rising progressively throughout the 120 min postprandial period. Relative to baseline, GLP-1 increased substantially following PRO ingestion at 30 min (*d*z = 1.72), 90 min (*d*z = 2.26), and 120 min (*d*z = 2.10). The CHO meal induced an initial rise that peaked at 30 min but declined thereafter, falling below FAT at 90 min (*t*_126_ = −2.91, *p* = 0.0043, *d*z = 0.60) and 120 min (*t*_123_ = −2.72, *p* = 0.0076, *d*z = 0.80). The FAT meal produced a delayed but sustained response that became apparent at 60 min and remained elevated throughout the observation period. By 120 min, GLP-1 concentrations in the PRO condition exceeded both CHO (*d*z = 1.83) and FAT (*d*z = 1.61), indicating markedly greater incretin stimulation following protein ingestion.

### 3.2. Appetite Control Hormone Outcomes

**Figure 7:**
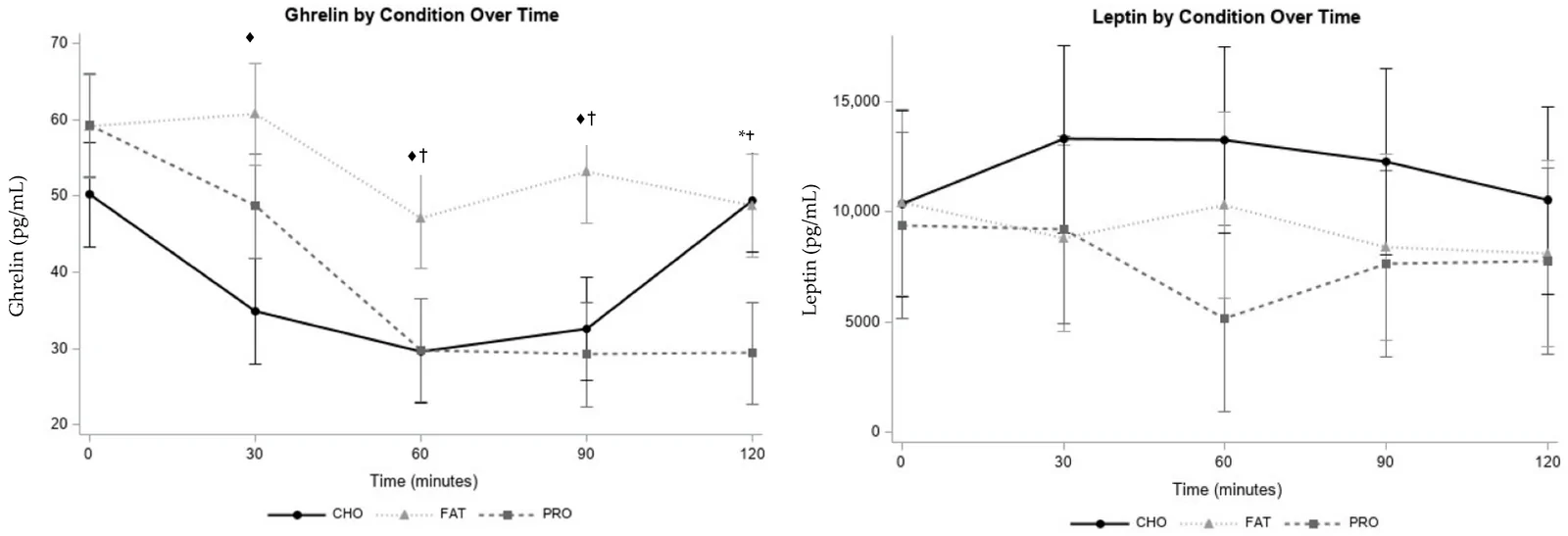
Concentrations of ghrelin and leptin over time. * CHO significantly different than PRO (*p* < 0.05). ♦ CHO significantly different than FAT (*p* < 0.05). † PRO significantly different than FAT (*p* < 0.05). Mean ± Standard Error.

Ghrelin and leptin displayed distinct postprandial responses (Figure 7). For ghrelin, both the PRO and CHO meals induced early suppression, although the CHO response reached its nadir sooner. Ghrelin concentrations subsequently increased following CHO between 90 and 120 min, whereas the PRO condition remained suppressed throughout the observation period. Relative to baseline, ghrelin following PRO decreased over the first 60 min and then stabilized, with moderate-to-large effect sizes through 90 min (*d*z = 0.66–0.85). At 120 min, ghrelin concentrations in the PRO condition were significantly lower than both CHO (*t*_153_ = 2.83, *p* = 0.0052, *d*z = 0.49) and FAT (*t*_153_ = 2.76, *p* = 0.0065, *d*z = 0.47). The FAT meal produced a delayed and attenuated effect, with no significant suppression evident at 30 min. By 120 min, FAT and CHO concentrations did not differ (*t*_153_ = 0.10, *p* = 0.9195, *d*z = 0.05).

Leptin concentrations exhibited modest postprandial variation with no significant effects of condition or time. None of the between-condition comparisons were significant (CHO vs. FAT: *t*_46.5_ = 0.87, *p* = 0.3861, *d*z = 0.11; CHO vs. PRO: *t*_46.6_ = 1.32, *p* = 0.1919, *d*z = 0.15; FAT vs. PRO: *t*_46.1_ = 0.45, *p* = 0.6541, *d*z = 0.12). Overall, leptin responses were characterized by small effect sizes and minimal temporal variation, indicating that leptin was largely insensitive to acute macronutrient composition across the 120 min postprandial period.

**Figure 8:**
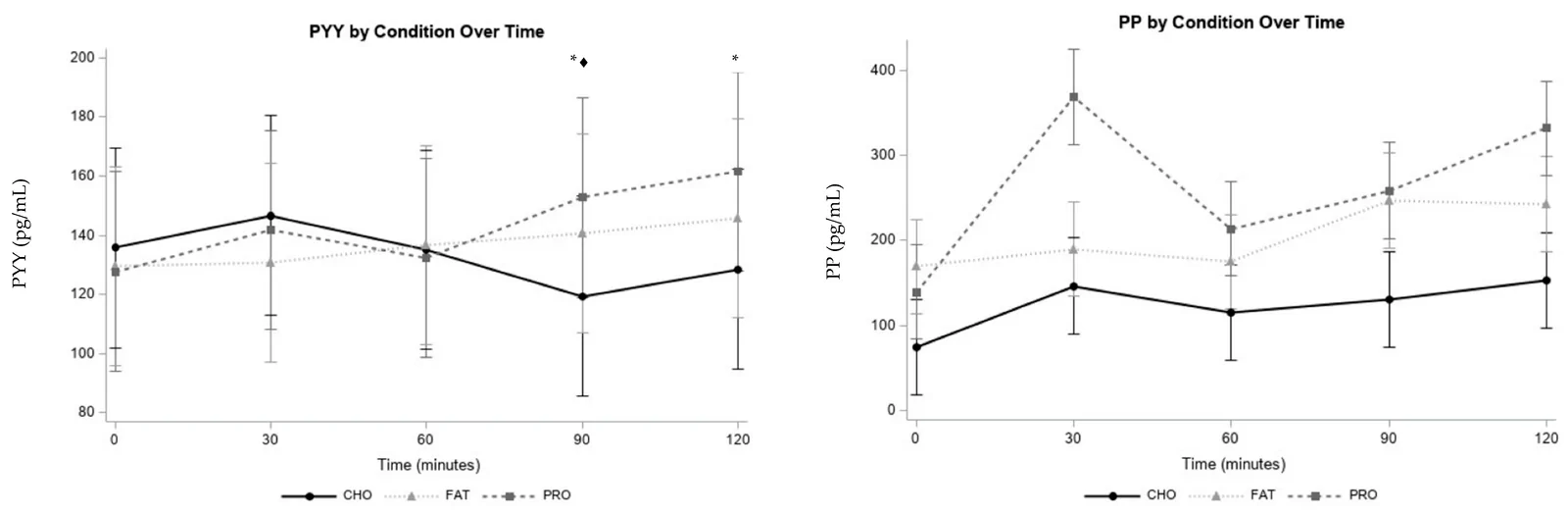
Concentrations of PP and PYY over time. * CHO significantly different than PRO (*p* < 0.05). ♦ CHO significantly different than FAT (*p* < 0.05). Mean ± Standard Error.

For PYY (Figure 8), no significant pairwise differences were observed between conditions during the first 60 min. At 90 min, CHO concentrations were lower than both PRO (*t*_255_ = −3.09, *p* = 0.0022, *d*z = 0.70) and FAT (*t*_254_ = −1.99, *p* = 0.0473, *d*z = 0.33). At 120 min, CHO remained lower than PRO (*t*_251_ = −3.13, *p* = 0.0020, *d*z = 0.54).

A significant main effect was observed for PP (Figure 8) across condition (*F*_2,46.6_ = 7.44, *p* = 0.0016) and time (*F*_4,268_ = 6.21, *p* < 0.0001), although there was no significant condition × time interaction (*F*_8,268_ = 1.66, *p* = 0.1094). Post hoc analyses indicated that PP concentrations were lower in the CHO condition than in both FAT (*t*_46.8_ = −2.25, *p* = 0.0294) and PRO (*t*_46.9_ = −3.84, *p* = 0.0004). PP concentrations also increased between 60 and 120 min across conditions (*t*_267_ = −2.73, *p* = 0.0068, *d*z = 0.54).

### 3.3. Inflammation Hormone Outcomes

**Figure 9:**
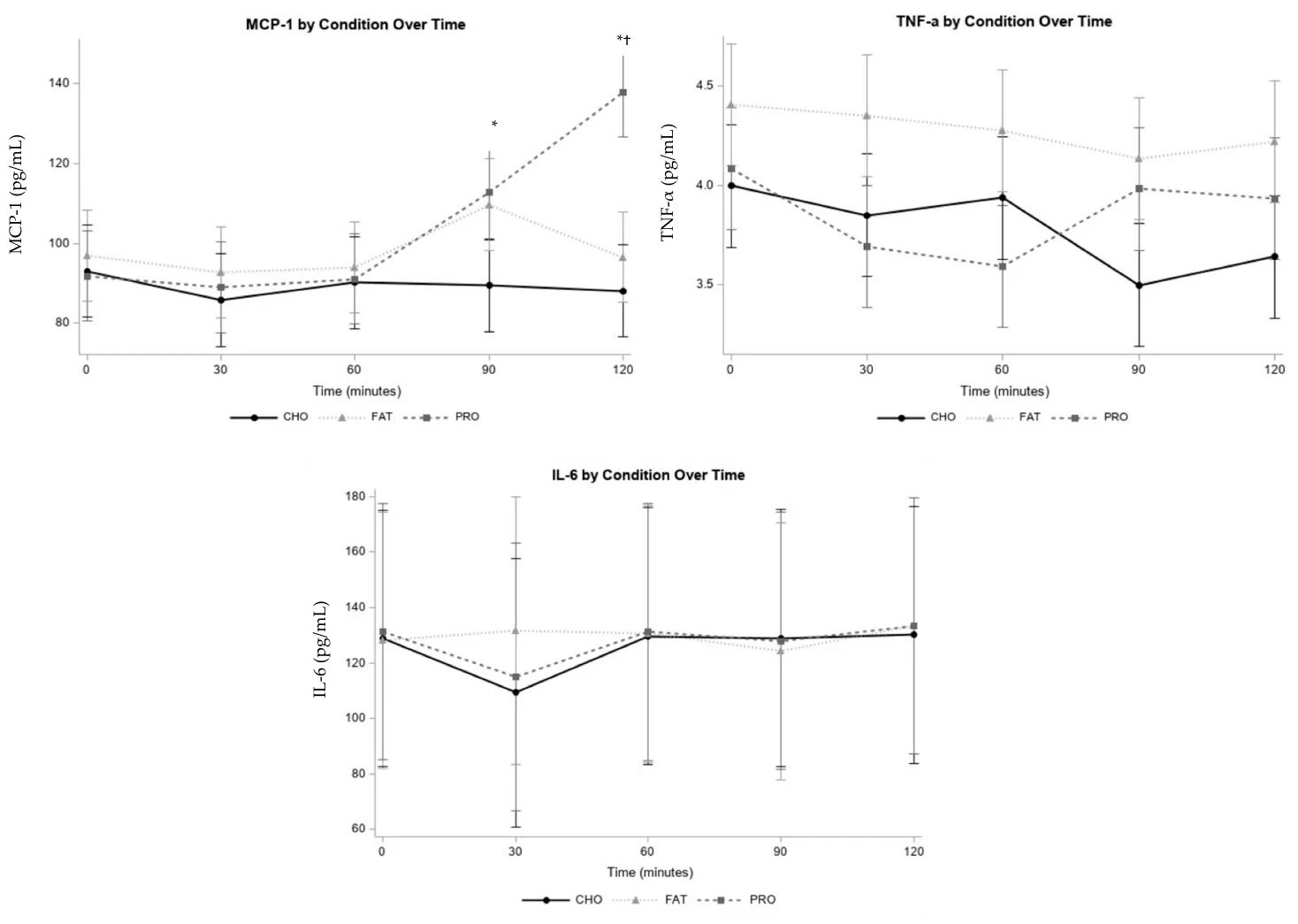
Concentrations of MCP-1, TNF-α and IL-6 over time. * CHO significantly different than PRO (*p* < 0.05). † PRO significantly different than FAT (*p* < 0.05). Mean ± Standard Error.

There was no significant effect of condition on MCP-1 during the first 60 min. However, the significant condition × time interaction (*F*_8,269_ = 2.33, *ηp*^2^ = 0.07, *p* = 0.0194) was driven by a rise in MCP-1 following PRO ingestion beginning at 90 min. Relative to baseline, MCP-1 increased in the PRO condition with a moderate effect size at 90 min (*d*z = 0.71). By 120 min, MCP-1 concentrations in the PRO condition were significantly higher than both FAT (*t*_125_ = 3.28, *p* = 0.0012, *d*z = 0.28) and CHO (*t*_125_ = 3.90, *p* = 0.0001, *d*z = 0.36). In contrast, no significant main or interactive effects were detected for IL-6 (all *p* ≥ 0.1459) or TNF-α (all *p* ≥ 0.1117; Figure 9).

## 4. Discussion

### 4.1. Glycemic Control Outcomes

While isolated macronutrient challenges elicited highly distinct endocrine patterns, glycemic responses remained stable following both pure protein and lipid ingestion. Dextrose intake (CHO) induced expected acute glycemic excursions and coordinated elevations in insulin, C-peptide, and amylin, characteristic of standard beta-cell activation [10,11,12]. Conversely, the lack of glycemic shifts following FAT or PRO suggests that single nutritional boluses do not destabilize immediate glucose levels in low-carbohydrate-adapted individuals. This lines up with prior investigations demonstrating relative postprandial glucose stability after fat or protein ingestion in healthy states [13,14].

Importantly, pure protein (PRO) acted as an exceptionally potent stimulus for both glucagon and GLP-1 secretion. Although a prominent rise in glucagon is traditionally linked with increased hepatic glucose output, glucose levels here remained completely unchanged within the 120 min postprandial period. This suggests that the acute aminogenic glucagon surge was counterbalanced by concurrent insulinotropic and incretin mechanisms. Specifically, the massive, parallel expansion of GLP-1—a known inhibitor of excessive glucagon action and gastric emptying—alongside modest increases in C-peptide and amylin likely acted to constrain endogenous glucose appearance [15]. Consequently, our findings indicate that acute protein-induced glucagon release does not exert an independent, uninhibited driving force on circulating blood glucose in this cohort, highlighting a preserved and resilient counter-regulatory network.

During the intervention sessions, macronutrient-specific hormonal profiles aligned with established physiological responses. Glucose rose sharply following the carbohydrate load, consistent with classic elevations in insulin [16], C-peptide [17], and amylin [18] and reflective of beta-cell activation observed in mixed-meal or glucose challenges [19]. In contrast, glucose remained stable in both the FAT and PRO conditions, demonstrating that neither of these macronutrients meaningfully elevated glycemia within the two-hour postprandial window. These findings are supported by Traianedes et al. who provided various amounts and types of dietary fat (safflower oil, olive oil, butter, or medium-chain triglycerides) and found that even when dietary fat composed over 50% of the calories for a meal, postprandial glucose and insulin concentrations were not elevated [20]. Likewise, multiple studies have demonstrated that ingestion of protein results in little or no increase in circulating glucose concentrations in healthy individuals [21,22,23]. Our work supports these previous works and demonstrates that this remains consistent in a group of individuals maintaining a low-carbohydrate diet.

The protein condition produced the largest rise in GLP-1 and glucagon, which is consistent with protein’s role as a potent stimulus of both hormones [24,25]. GLP-1 is also known to dampen glucagon secretion [26] which could be a potential mechanism explaining why the individuals in this study do not have excessive glucagon and, therefore, elevated glucose concentrations. Amylin and C-peptide also increased following PRO, which may have further contributed to stable glucose concentrations by slowing gastric emptying [27] and supporting insulin secretion [28,29]. The lack of a glucose rise following PRO suggests that the glucagon response alone did not overcome the insulinotropic and incretin-mediated mechanisms that suppress hepatic glucose output, at least in the short term.

Although GIP is a well-established incretin hormone that rises sharply in response to carbohydrate intake [15], the present findings showed distinct separation between conditions. The CHO drink elicited a robust and rapid GIP peak, whereas the FAT and PRO drinks produced more delayed and muted responses. GLP-1, on the other hand, rose most prominently in the PRO condition, which is consistent with emerging evidence that protein is a stronger stimulus for GLP-1 secretion than carbohydrates [30,31]. Together, the incretin results show that macronutrient composition distinctly shapes postprandial glucose-regulating hormone profiles, yet in this cohort none of these hormonal signatures produced an exaggerated glycemic rise following FAT or PRO.

Beyond pancreatic and gut-derived endocrine signaling, the acute response to the carbohydrate bolus must be interpreted through tissue-level glucose handling kinetics. Following prolonged carbohydrate restriction, peripheral tissues undergo a well-characterized metabolic shift characterized by regular downregulation of carbohydrate-disposal pathways to prioritize lipid oxidation. This adaptive state, often described as “adaptive glucose sparing,” serves to preserve limited glucose availability for obligatory glycolytic tissues [32].

At the cellular level, this state is mediated by reduced glycolytic enzyme expression, diminished flux through the pyruvate dehydrogenase (PDH) complex, and altered glucose transporter 4 (GLUT4) translocation dynamics in skeletal muscle [33,34]. When a substantial carbohydrate load is acutely introduced into this lipid-adapted framework, the peripheral clear rate is transiently constrained by this downregulation of glycolytic capacity [35]. This physiological inertia explains why blood glucose rose sharply during the CHO condition, peaking at over 160 mg/dL, despite the preservation of a robust, fully competent insulin and C-peptide response. The delayed clearing curve represents the time required for acute insulin concentrations to re-activate the PDH complex and recruit GLUT4 transporters to the cell membrane, rather than reflecting structural beta-cell dysfunction or pathological insulin resistance. This distinction is critical, as it frames the post-carbohydrate glycemic excursion as a normal, temporary consequence of metabolic specialization rather than an underlying metabolic disorder.

In sum, while the hypothesis that glucagon contributes to elevated glucose remains physiologically plausible, our data did not demonstrate that glucagon independently drives higher glucose in this group of individuals who consume a low-carbohydrate diet. These results suggest a more complex interplay between fasting physiology, dietary patterns, and counter-regulatory hormones than initially expected.

### 4.2. Objective Appetite Control Outcomes

Macronutrients differentially influence appetite-regulating hormones, and the current study confirmed several well-established physiological patterns. Ghrelin, an orexigenic hormone that typically decreases in response to nutrient ingestion [36], showed suppression following both the CHO and PRO conditions. Ghrelin remained most suppressed after the PRO load, suggesting a potentially stronger satiety signal compared to CHO or FAT. The delayed and modest response to the FAT condition aligns with prior findings that dietary fat has a weaker impact on early postprandial ghrelin suppression [37].

Leptin, a longer-term signal of energy sufficiency and adiposity, exhibited minimal sensitivity to acute macronutrient manipulation in the present study. Despite numerically lower postprandial leptin concentrations following PRO compared with CHO, particularly at later time points, these differences were not significant after correction for multiple comparisons, suggesting that short-term leptin dynamics are relatively resistant to meal-induced perturbations. This finding is consistent with the established physiology of leptin, which is primarily regulated by chronic energy balance and fat mass rather than acute nutrient ingestion [38]. Prior studies similarly report limited or delayed postprandial leptin responses following single meals, even when macronutrient composition differs substantially [39,40]. Collectively, the lack of a pronounced leptin response across conditions supports the interpretation that leptin is unlikely to mediate the acute appetite-related effects observed with PRO or CHO in this context and that other gut- and pancreas-derived hormones may play a more prominent role in short-term postprandial appetite regulation.

The incretin hormones GIP and GLP-1 also contribute to appetite regulation. GLP-1 is particularly known for its role in satiety and delayed gastric emptying [41]. The large GLP-1 response after PRO may reflect a meaningful satiety effect, consistent with past work demonstrating greater fullness after high-protein meals [42]. Conversely, CHO produced the highest GIP response. These findings are consistent with previous work from Deru et al. [43] which demonstrated that a high-carbohydrate protein shake elicited a large spike in GIP when used to break a fast and that a low-carbohydrate protein shake also increased GIP, but to a lesser extent.

PYY, an anorexigenic peptide secreted from the distal gut, typically rises after food intake and also contributes to satiety [44]. In the current study, PYY concentrations were lower in the CHO condition at later time points compared to FAT and PRO, suggesting that protein and fat may elicit stronger or more prolonged satiety signals in the postprandial period. Pancreatic polypeptide (PP), another peptide associated with meal termination and reduced food intake [45], showed overall lower concentrations in the CHO condition, which is also supported by the current literature.

Together, these appetite-related outcomes suggest that protein produced the strongest overall satiety hormone pattern, whereas carbohydrate produced the least robust suppression of hunger-promoting signals. Although appetite was not measured behaviorally, the hormonal responses provide physiological context that may help explain differences in subjective hunger observed in other macronutrient studies.

### 4.3. Inflammatory Hormone Outcomes

Inflammatory cytokines responded minimally to acute macronutrient intake. IL-6 and TNF-α did not differ between conditions over time. This is consistent with work showing that low-grade inflammatory markers often require prolonged or repeated stimuli to exhibit measurable changes and may be less sensitive to acute macronutrient boluses [46].

MCP-1, however, demonstrated a unique delayed increase following the PRO condition, beginning at 90 min and peaking at 120 min. As a chemokine involved in monocyte recruitment and inflammatory cell trafficking, MCP-1 concentrations typically decrease during fasting [46] but can increase postprandially. However, the literature remains mixed regarding which macronutrients elicit the strongest response [47,48,49,50]. In the present study, the selective postprandial MCP-1 increase after whey protein may reflect the acute influx of branched-chain amino acids (BCAAs), particularly leucine—a well-established activator of nutrient-sensitive mTORC1 signaling.

Importantly, the relationship between amino acids, mTORC1, and inflammation is context-dependent. In metabolically healthy tissue, acute amino acid signaling primarily reflects nutrient sensing and protein synthesis activation. In contrast, chronic BCAA elevations in obesity or insulin-resistant states are associated with impaired metabolic signaling and low-grade inflammation. For instance, Newgard et al. identified a BCAA-related metabolic signature that distinguishes individuals with obesity from lean individuals and associates with insulin resistance [47], while Lynch and Adams reviewed evidence linking BCAAs and mTOR signaling to metabolic dysfunction in insulin-resistant states [48].

Several mechanisms could plausibly explain this observed MCP-1 response, though they should be interpreted as hypothesis-generating rather than definitive. First, acute amino acid availability may activate mTORC1-dependent nutrient-sensing pathways, which can interact with inflammatory signaling networks. Second, MCP-1 expression is regulated by canonical inflammatory transcriptional pathways, including NF-κB—a central regulator of inflammatory gene transcription linked to increased mediator and chemokine expression [49,50]. Third, because participants varied widely in BMI, the MCP-1 response may have been influenced by underlying differences in adiposity or insulin resistance, which substantially alter BCAA metabolism and inflammatory signaling. Consequently, the postprandial rise in MCP-1 should not be interpreted as evidence that whey protein produced a generalized inflammatory response. Rather, it likely represents a transient chemokine signal related to rapid amino acid absorption, nutrient sensing, and host metabolic status.

To move beyond an exclusively endocrine-centered interpretation, glycemic control and incretin responses should be viewed through a systematic lens that integrates the gut microbiota and its downstream metabolites. Dietary carbohydrate restriction fundamentally shifts the gastrointestinal ecosystem away from carbohydrate fermentation toward protein and lipid utilization. For example, ketogenic diets alter human gut microbial structure in a manner distinct from standard high-fat diets, causing reproducible reductions in *Bifidobacterium* species driven in part by the systemic presence of ketone bodies [51]. Similarly, high-protein, low-carbohydrate diets reduce fecal butyrate levels and deplete the *Roseburia*/*Eubacterium rectale* group, which are key carbohydrate-fermenting bacteria [52]. Furthermore, ketogenic diets, but not free-sugar restriction alone, induce distinct microbial shifts, confirming that absolute carbohydrate restriction produces ecosystem-wide effects beyond simple sugar reduction [33].

These microbial shifts carry clear metabolic implications. Gut-derived short-chain fatty acids (SCFAs), such as acetate, propionate, and butyrate, directly interact with host pathways regulating glucose homeostasis, intestinal barrier integrity, and low-grade inflammatory tone [53,54]. Mechanistically, SCFAs act as signaling ligands that stimulate intestinal L-cell secretion of GLP-1 and PYY [55], which directly relates to the incretin and appetite outcomes tracked in the present study. Because gut microbiota composition and fecal SCFA profiles were not measured in this cohort, these pathways remain speculative.

### 4.4. Strengths and Limitations

A notable strength of this study is its randomized crossover design, which enables each participant to act as their own control, thereby minimizing inter-individual variability and enhancing internal validity. Moreover, the incorporation of continuous glucose monitoring (CGM) offers high temporal resolution of glycemic dynamics that would likely be missed with intermittent sampling approaches. In addition, the use of multiplex hormonal assays allowed for the simultaneous quantification of multiple peptides involved in glycemic regulation, appetite signaling, and inflammation, providing a more integrated view of the physiological response within a single experimental framework.

Several limitations warrant consideration, though they do not substantially detract from the primary interpretations. First, while the overall sample size was sufficient to detect within-subject effects for the major hormones, the subset of participants with viable CGM data (*n* = 16 of 24) introduces potential constraints on statistical power for glucose-specific outcomes and may affect representativeness. That said, crossover designs inherently improve efficiency, and the consistency of observed within-subject trends mitigates concern that the reduced CGM sample meaningfully biases the primary conclusions. Nonetheless, future studies with larger samples and more complete CGM datasets would strengthen external validity.

Second, the BMI range of the study sample was broad, extending from normal weight to severe obesity. Although BMI was included as a covariate in the mixed-effects models, this adjustment may not fully account for differences in adiposity-related insulin resistance, beta-cell function, hepatic insulin sensitivity, or incretin responsiveness. These factors could influence fasting insulin, C-peptide, glucagon, GIP, GLP-1, and postprandial glucose handling independent of low-carbohydrate adherence [4,5,56,57]. Because insulin sensitivity was not directly assessed, we could not determine whether obesity or insulin resistance modified the hormonal responses to each macronutrient bolus. Future studies should stratify participants by BMI or insulin-sensitivity status and include direct measures such as HOMA-IR, oral glucose tolerance testing, or hyperinsulinemic–euglycemic clamp techniques.

Third, the 2-week dietary run-in period must be interpreted strictly as a minimum eligibility threshold for early-phase adaptation, rather than evidence of a complete, steady-state metabolic transition. While prominent shifts in postprandial signaling and baseline substrate choice may manifest within 14 days, complete multi-system metabolic keto-adaptation, including full stability of hepatic gluconeogenic flux, tissue-level lipid processing, and gut microbial remodeling, may require a longer period. Thus, our acute postprandial profiles capture an early adaptive phase that may differ from individuals established on long-term low-carbohydrate lifestyles.

Forth, the acute nature of the intervention limits inference regarding longer-term regulatory processes. The study was designed to isolate immediate postprandial responses under tightly controlled conditions, which is appropriate for mechanistic investigation but does not address chronic adaptations in glucose or hormone regulation. Importantly, acute responses are often predictive of longer-term trends, though this relationship is not absolute and should be interpreted cautiously.

Fifth, reliance on self-reported dietary adherence outside of the laboratory introduces the possibility of variability in baseline metabolic conditions across testing sessions. While this is a common and largely unavoidable constraint in free-living nutrition studies, the crossover design again helps to mitigate this concern, as each participant’s responses are compared relative to their own baseline physiology rather than between individuals.

Sixth, the use of single-macronutrient boluses, while advantageous for isolating nutrient-specific physiological effects, does not reflect typical mixed-meal consumption patterns. As such, ecological validity is somewhat limited. However, this reductionist approach is appropriate for disentangling macronutrient-driven mechanisms, which would be more difficult to interpret in the context of mixed meals. These findings therefore provide a foundational understanding that can inform more applied, real-world feeding studies.

An additional limitation is the large number of outcomes evaluated, which required multiple models and repeated contrasts. We did not apply a global multiplicity correction across all outcomes. In this small exploratory crossover study, such corrections would be highly conservative and would substantially increase the risk of Type II error for biologically meaningful responses. To reduce this concern and support interpretation, we emphasized a priori planned comparisons, effect sizes, and the mechanistic plausibility of each finding, as well as the consistency, timing, and physiological coherence of each response. Nevertheless, Type I error remains possible, particularly for less-robust findings.

Finally, the present study lacks the tracking of circulating ketone bodies, specifically beta-hydroxybutyrate (BHB). In individuals adhering to a low-carbohydrate intake, BHB functions not merely as an alternative fuel source but as a potent epigenetic and metabolic signaling molecule. BHB directly modulates host physiology by activating the GPR109A receptor, directly inhibiting histone deacetylase activity, and dampening innate pro-inflammatory pathways [58]. Because circulating ketone concentrations were not quantified in this protocol, we cannot determine the extent to which variable baseline ketosis may have cross-regulated beta-cell insulin secretion or inflammatory biomarker kinetics across our participants. Tracking blood BHB levels must be a standard requirement in future low-carbohydrate trials.

### 4.5. Future Directions

Future research should examine whether chronic protein-induced glucagon elevations contribute to longer-term glycemic patterns in low-carbohydrate dieters. Studies that directly compare individuals who experience paradoxical glucose elevations to those who do not would help clarify the underlying physiology. Integrating measures of hepatic glucose output and insulin sensitivity could further clarify the roles of gluconeogenesis, glucagon signaling, and incretin activity. Finally, longer observation windows and mixed-meal paradigms may yield a more complete understanding of how real-world dietary patterns influence glucose and hormonal regulation.

## 5. Conclusions

In adults adhering to a low-carbohydrate diet, isolated carbohydrate, protein, and fat boluses elicited distinct endocrine responses despite markedly different effects on postprandial glycemia. Carbohydrate produced the greatest increases in glucose, insulin, C-peptide, amylin, and GIP, whereas protein elicited the largest glucagon and GLP-1 responses while maintaining glucose concentrations comparable to fat. These findings demonstrate that substantial differences in postprandial hormone secretion do not necessarily translate into differences in short-term glycemic control and highlight the coordinated actions of pancreatic and incretin hormones in preserving glucose homeostasis.

Protein also produced the most favorable appetite-regulating hormonal profile, characterized by sustained ghrelin suppression, robust GLP-1 secretion, and higher concentrations of PYY and pancreatic polypeptide relative to carbohydrate. In contrast, carbohydrate elicited the least robust satiety-related hormonal response while producing the largest glycemic excursion. Inflammatory responses were minimal across all conditions, with only a modest increase in MCP-1 following protein ingestion and no significant changes in IL-6 or TNF-α.

Overall, these findings demonstrate that macronutrient composition profoundly influences the endocrine pathways governing glycemic regulation and appetite control in low-carbohydrate-adapted individuals, with protein eliciting a hormonal profile consistent with enhanced satiety while maintaining glycemic stability. Future studies should determine whether these acute endocrine responses translate into meaningful long-term differences in appetite regulation, energy intake, and metabolic health outcomes.

## Figures and Tables

**Table 1:** Demographic characteristics of participants.

	Male (*n* = 11)		Female (*n* = 13)		Cumulative (*n* = 24)	
	Mean	SD	Mean	SD	Mean	SD
**Age (years)**	42.6	10.5	41.2	12.7	41.9	11.6
**BMI (kg/m^2^)**	29.7	6.6	27.1	6.9	28.3	6.8
**BF %**	27.3	9.5	37.1	8.4	32.6	9.9
**Ethnicity**	*n*	%	*n*	%	*n*	%
**Caucasian**	9	82	13	100	22	92
**Hispanic/Latino**	1	9	-	-	1	4
**Pacific Islander**	1	9	-	-	1	4

**Table 2:** Hormone concentrations at each time point separated by condition.

Hormone	Condition	0 min	30 min	60 min	90 min	120 min	F	*p*
Glucagon	CHO	88.8 (21.5)	65.9 (21.8) ^a^	54.6 (21.5) ^a^	51.9 (21.5) ^a^	80.1 (21.5) ^a^	18.71	<0.0001
	PRO	89.4 (21.3)	270.9 (21.5) ^b^	264.1 (21.3) ^b^	316.3 (21.7) ^b^	268.2 (21.3) ^b^		
	FAT	87.4 (21.3)	85.5 (21.3) ^a^	90.1 (21.3) ^a^	87.7 (21.5) ^a^	93.9 (21.3) ^a^		
Insulin	CHO	2449.3 (651.1)	4067.1 (652.5) ^a^	4767.2 (651.2) ^a^	3653.0 (651.2)	2916.6 (651.2) ^a^	7.56	<0.0001
	PRO	2485.4 (649.9)	3155.9 (651.2) ^b^	3216.8 (649.9) ^b^	3309.7 (652.4)	3155.3 (649.9) ^a^		
	FAT	2484.9 (649.9)	2487.3 (649.9) ^c^	2499.6 (649.9) ^c^	2457.6 (651.1)	2490.1 (649.9) ^b^		
Amylin	CHO	13.3 (3.1)	41.8 (3.2) ^a^	50.9 (3.1) ^a^	39.0 (3.1) ^a^	28.5 (3.1) ^a^	18.85	<0.0001
	PRO	12.6 (3.1)	24.3 (3.1) ^b^	24.5 (3.1) ^b^	28.1 (3.2) ^b^	25.0 (3.1) ^a^		
	FAT	13.4 (3.1)	12.8 (3.1) ^c^	13.1 (3.1) ^c^	11.9 (3.1) ^c^	12.0 (3.1) ^b^		
C-Peptide	CHO	1277.4 (312.1)	4143.5 (316.9) ^a^	5524.2 (312.7) ^a^	4702.8 (312.7) ^a^	3198.7 (312.7) ^a^	7.98	<0.0001
	PRO	1222.0 (308.1)	2442.0 (312.1) ^b^	2438.6 (308.1) ^b^	2846.0 (316.3) ^b^	2528.4 (308.1) ^a^		
	FAT	1250.6 (308.1)	1169.1 (308.1) ^c^	1221.6 (308.1) ^c^	1095.7 (312.1) ^c^	1087.1 (308.1) ^b^		
GIP	CHO	102.1 (57.7)	667.8 (58.7) ^a^	764.0 (57.8) ^a^	596.2 (57.8)	474.4 (57.8) ^a^	8.46	<0.0001
	PRO	118.8 (56.8)	569.6 (57.7) ^a^	451.5 (56.8) ^b^	510.2 (58.5)	467.9 (56.8) ^a,b^		
	FAT	136.0 (56.8)	182.6 (56.8) ^b^	374.9 (56.8) ^b^	368.2 (57.7)	444.8 (56.8) ^b^		
GLP-1	CHO	201.4 (39.4)	358.2 (40.2) ^a^	266.1 (39.5) ^a^	179.3 (39.5) ^a^	159.5 (39.5) ^a^	17.38	<0.0001
	PRO	209.7 (38.8)	471.0 (39.4) ^b^	484.6 (38.8) ^b^	626.7 (40.0) ^b^	660.2 (38.8) ^b^		
	FAT	198.2 (38.8)	207.5 (38.8) ^c^	289.4 (38.8) ^a^	324.6 (39.4) ^c^	293.9 (38.8) ^c^		
Ghrelin	CHO	50.1 (6.8)	34.9 (6.9) ^a^	29.6 (6.8) ^a^	32.6 (6.8) ^a^	49.5 (6.8) ^a^	3.91	0.0002
	PRO	59.3 (6.7)	48.7 (6.8) ^a,b^	29.8 (6.7) ^a^	29.2 (6.9) ^a^	29.3 (6.7) ^b^		
	FAT	59.1 (6.7)	60.7 (6.7) ^b^	47.1 (6.7) ^b^	53.2 (6.8) ^b^	48.7 (6.7) ^a^		
Leptin	CHO	10,376 (4228.7)	13,284 (4245.9)	13,262 (4233.0)	12,250 (4233.0)	10,507 (4233.0)	1.32	0.2350
	PRO	9351.6 (4216.5)	9176.7 (4228.6)	5143.6 (4216.5)	7644.9 (4241.0)	7761.1 (4216.5)		
	FAT	10,438 (4216.5)	8813.9 (4216.5)	10,323 (4216.5)	8376.4 (4228.3)	8121.6 (4216.5)		
PP	CHO	74.3 (56.0)	146.5 (56.6)	115.1 (56.0)	130.7 (56.0)	153.0 (56.0)	1.66	0.1094
	PRO	139.5 (55.4)	368.7 (56.0)	213.6 (55.4)	258.1 (56.5)	331.7 (55.4)		
	FAT	169.4 (55.4)	189.9 (55.4)	175.1 (55.4)	246.8 (56.0)	242.5 (55.4)		
PYY	CHO	135.7 (33.7)	146.6 (33.8)	135.1 (33.7)	119.4 (33.7) ^a^	128.5 (33.7) ^a^	2.65	0.0082
	PRO	127.7 (33.7)	141.8 (33.7)	132.2 (33.7)	152.7 (33.8) ^b^	161.6 (33.7) ^b^		
	FAT	129.6 (33.7)	130.6 (33.7)	136.5 (33.7)	140.7 (33.7) ^b^	145.8 (33.7) ^a,b^		
MCP-1	CHO	93.1 (11.4)	85.8 (11.6)	90.2 (11.5)	89.4 (11.5)	88.2 (11.5) ^a^	2.33	0.0194
	PRO	91.8 (11.3)	89.0 (11.4)	91.1 (11.3)	112.8 (11.6)	137.8 (11.3) ^b^		
	FAT	96.9 (11.3)	92.7 (11.3)	94.0 (11.3)	109.7 (11.4)	96.5 (11.3) ^a^		
IL-6	CHO	128.9 (46.3)	109.3 (48.5)	129.7 (46.3)	129.0 (46.4)	130.1 (46.3)	0.72	0.6730
	PRO	131.4 (46.3)	114.9 (48.4)	131.2 (46.3)	127.9 (46.4)	133.4 (46.3)		
	FAT	128.2 (46.3)	131.6 (48.3)	130.5 (46.3)	124.3 (46.4)	133.3 (46.3)		
TNF-α	CHO	4.0 (0.3)	3.8 (0.3)	3.9 (0.3)	3.5 (0.3)	3.6 (0.3)	1.544	0.1423
	PRO	4.1 (0.3)	3.7 (0.3)	3.6 (0.3)	4.0 (0.3)	3.9 (0.3)		
	FAT	4.4 (0.3)	4.3 (0.3)	4.3 (0.3)	4.1 (0.3)	4.2 (0.3)		

Mean (SD) hormone concentrations are reported in pg/mL. ^a,b,c^ Concentrations with different superscript letters in the same row are significantly different from each other.

## Data Availability

The original data sets are publicly available through the Open Science Framework website at the following URL: https://osf.io/c35sa/overview?view_only=5d606a89b16c426d9460e3ebd74d5f09. Accessed on 10 July 2026.
